# LY2405319, an Engineered FGF21 Variant, Improves the Metabolic Status of Diabetic Monkeys

**DOI:** 10.1371/journal.pone.0065763

**Published:** 2013-06-18

**Authors:** Andrew C. Adams, Carolyn A. Halstead, Barbara C. Hansen, Armando R. Irizarry, Jennifer A. Martin, Sharon R. Myers, Vincent L. Reynolds, Holly W. Smith, Victor J. Wroblewski, Alexei Kharitonenkov

**Affiliations:** 1 Lilly Research Laboratories, Eli Lilly and Company, Indianapolis, Indiana, United States of America; 2 Center for Preclinical Research, University of South Florida, Tampa, Florida, United States of America; The University of Hong Kong, Hong Kong

## Abstract

Fibroblast growth factor 21 (FGF21) is a novel metabolic regulator that represents a promising target for the treatment of several metabolic diseases. Administration of recombinant wild type FGF21 to diabetic animals leads to a dramatic improvement in glycaemia and ameliorates other systemic measures of metabolic health. Here we report the pharmacologic outcomes observed in non-human primates upon administration of a recently described FGF21 analogue, LY2405319 (LY). Diabetic rhesus monkeys were treated subcutaneously with LY once daily for a period of seven weeks. The doses of LY used were 3, 9 and 50 mg/kg each delivered in an escalating fashion with washout measurements taken at 2, 4, 6 and 8 weeks following the final LY dose. LY therapy led to a dramatic and rapid lowering of several important metabolic parameters including glucose, body weight, insulin, cholesterol and triglyceride levels at all doses tested. In addition, we observed favorable changes in circulating profiles of adipokines, with increased adiponectin and reduced leptin indicative of direct FGF21 action on adipose tissue. Importantly, and for the first time we show that FGF21 based therapy has metabolic efficacy in an animal with late stage diabetes. While the glycemic efficacy of LY in this animal was partially attenuated its lipid lowering effect was fully preserved suggesting that FGF21 may be a viable treatment option even in patients with advanced disease progression. These findings support continued exploration of the FGF21 pathway for the treatment of metabolic disease.

## Introduction

The conventional approach to discover novel drugs for diabetes mellitus is traditionally centered on agents able to lower blood glucose with little to no attention paid to other measures of metabolic health. However, long term clinical outcomes with the molecules developed via this methodology have been mixed at best. This has led to a steady though guarded rise in attention towards novel so-called “glucose plus” targets that act via multifaceted mechanisms thus offering additional metabolic benefits beyond improvements in glycemic control [1], [2]. One example of this new class of molecules is fibroblast growth factor 21 (FGF21) [3], [4], which recently emerged as a promising agent able to simultaneously correct many if not all abnormalities in metabolically compromised animals [5]–[7]. Indeed, administration of recombinant wild type FGF21 to diabetic rodents leads to a rapid and robust lowering of hyperglycemia, improvements in circulating/tissue lipid levels and reduced body weight [8]–[11]. The attractiveness of FGF21 based preclinical pharmacology as a superior “glucose plus” agent has already inspired initial efforts to optimize and evaluate re-engineered FGF21 analogues in animal models of metabolic disease [12]–[14].

We have previously demonstrated a diverse spectrum of pharmacologic actions in diabetic monkeys following treatment with human recombinant FGF21 [15]. Nevertheless, the advancement of native FGF21 as a drug candidate is faced with significant development challenges, most notably due to biopharmaceutical issues of the wild type molecule [14]. Recently, we described the structure, biopharmaceutical properties and nonclinical efficacy profile of an FGF21 based clinical candidate, LY2405319 (LY) [14]. In brief, to facilitate a once-daily dosing profile, in a preserved, multi-use formulation, an additional disulfide bond was introduced in FGF21 through Leu118Cys and Ala134Cys mutations. FGF21 was further optimized by deletion of the four N-terminal amino acids, His-Pro-Ile-Pro (HPIP), which are subject to proteolytic cleavage in the native molecule. In addition, to eliminate an O-linked glycosylation site in yeast a Ser167Ala mutation was introduced, allowing large-scale homogenous protein production in *Pichia pastoris*.

Following the initial studies in rodents [14] we went on to evaluate the pharmacokinetic and pharmacodynamic properties of LY following its administration for 7 weeks in a dose-escalating fashion in a well-defined group of diabetic rhesus monkeys (*Macaca mulatta*).

In this report we show that LY administration led to dramatic and rapid improvements in metabolic parameters including lowering of circulating glucose/insulin, ameliorated plasma lipid profiles and reduced body weight. These effects were observed in the presence of treatment-emergent LY antibodies. Importantly, in an animal with advanced disease progression, the effects of LY on circulating lipids and body weight were readily present even though its glycemic efficacy was significantly attenuated. In conclusion, our new data with LY, an engineered FGF21 variant, support further exploration of FGF21-based therapies for the treatment of metabolic disorders, including studies in patients at the advanced stages of disease development.

## Methods

### LY Expression and Purification

LY was expressed and purified from *Pichia pastoris* as described [14], and formulated in bacteriostatic NaCl (0.9%).

### Animals

All animal protocols in this study were approved by the Eli Lilly and Co. Animal Use and Care Committee. All monkeys were single housed in stainless steel cages for the duration of the protocol to allow for measurement of food intake as previously described (Hansen BC, 2012, Animal Models in Diabetes Research, Methods in Molecular Biology), water was provided ad libitum. Monkeys were housed in a temperature controlled facility (21°C ±3°C) with a 12 h light/dark cycle. Animals were provided with environmental enrichment and were routinely monitored for signs of illness or distress by the veterinary care staff throughout the duration of the study. However, at the time of cessation of dosing none of the animals had shown any physical signs of distress or discomfort. None of the animals were sacrificed at any point during the study.

To establish a metabolic baseline, vehicle was administered daily by subcutaneous injection for 6 to 15 consecutive days to a total of 6 diabetic rhesus monkeys of either sex that had no prior exposure to any FGF21 based molecule. Subsequently, daily subcutaneous injections of LY were administered in a dose-escalating fashion (3, 9, and 50 mg/kg) for a total of 7 weeks (2 weeks for the 3 and 9 mg/kg treatment period, and 3 weeks for the 50 mg/kg treatment period) followed by a 60-day follow-up period (no injections). A 1- to 2-day washout period was included after each of the three LY treatment periods to allow for washout of the drug prior to drawing samples for assessment of immunogenicity. Volumes administered ranged from 0.06 mL/kg to 1 mL/kg. Sites of subcutaneous administration were rotated among multiple sites on the thorax/abdomen (most commonly the lateral sides and anterior surface) and sites were recorded for each dose.

During each period, the following parameters were monitored as functional biomarkers of LY pharmacodynamic activity: daily fed blood glucose and food intake, weekly body weight, bi-weekly fasted insulin, leptin, and adiponectin, and plasma lipids at the end of each treatment period.

**Figure 1:**
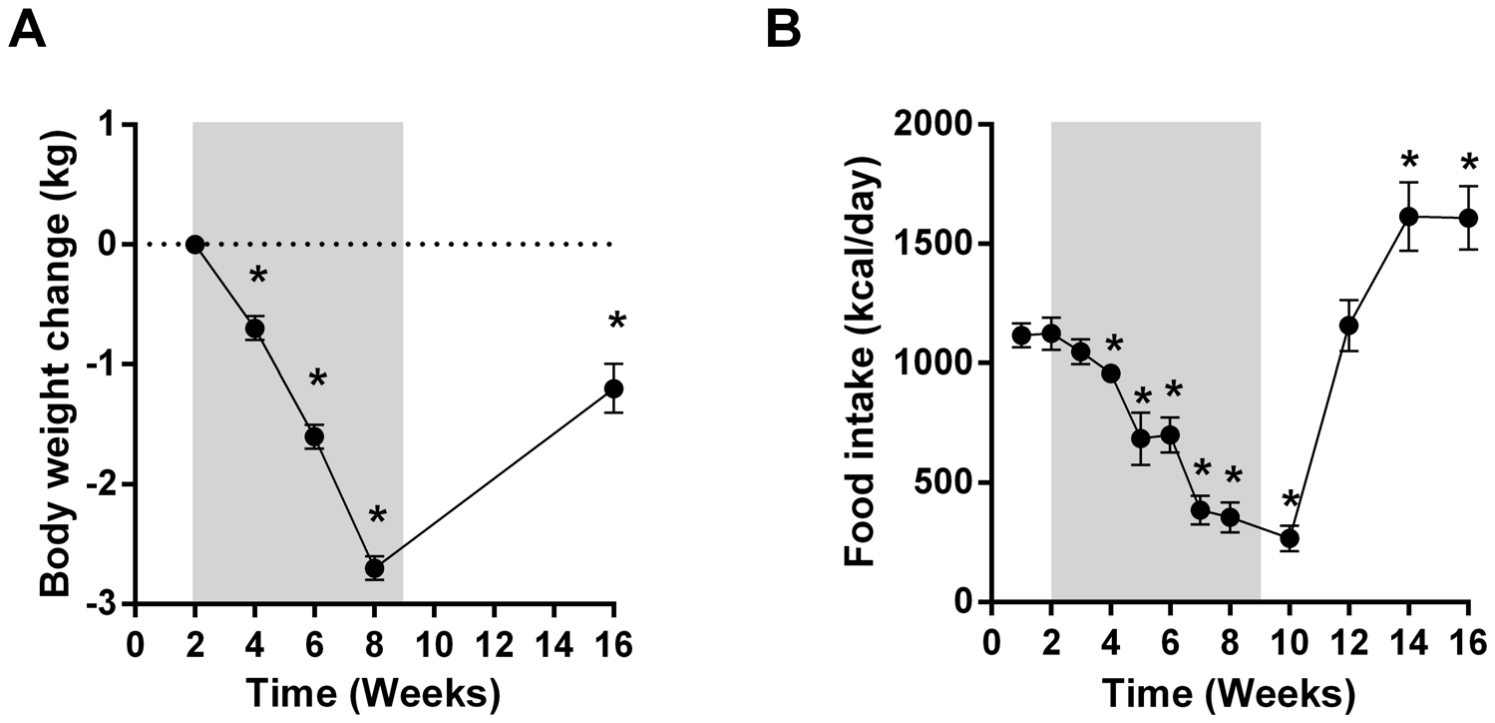
Effects of administration of a dose escalation of LY2405319 on body mass (A) and food intake (B). Differences considered significant (p<0.05) are denoted by *.

**Figure 2:**
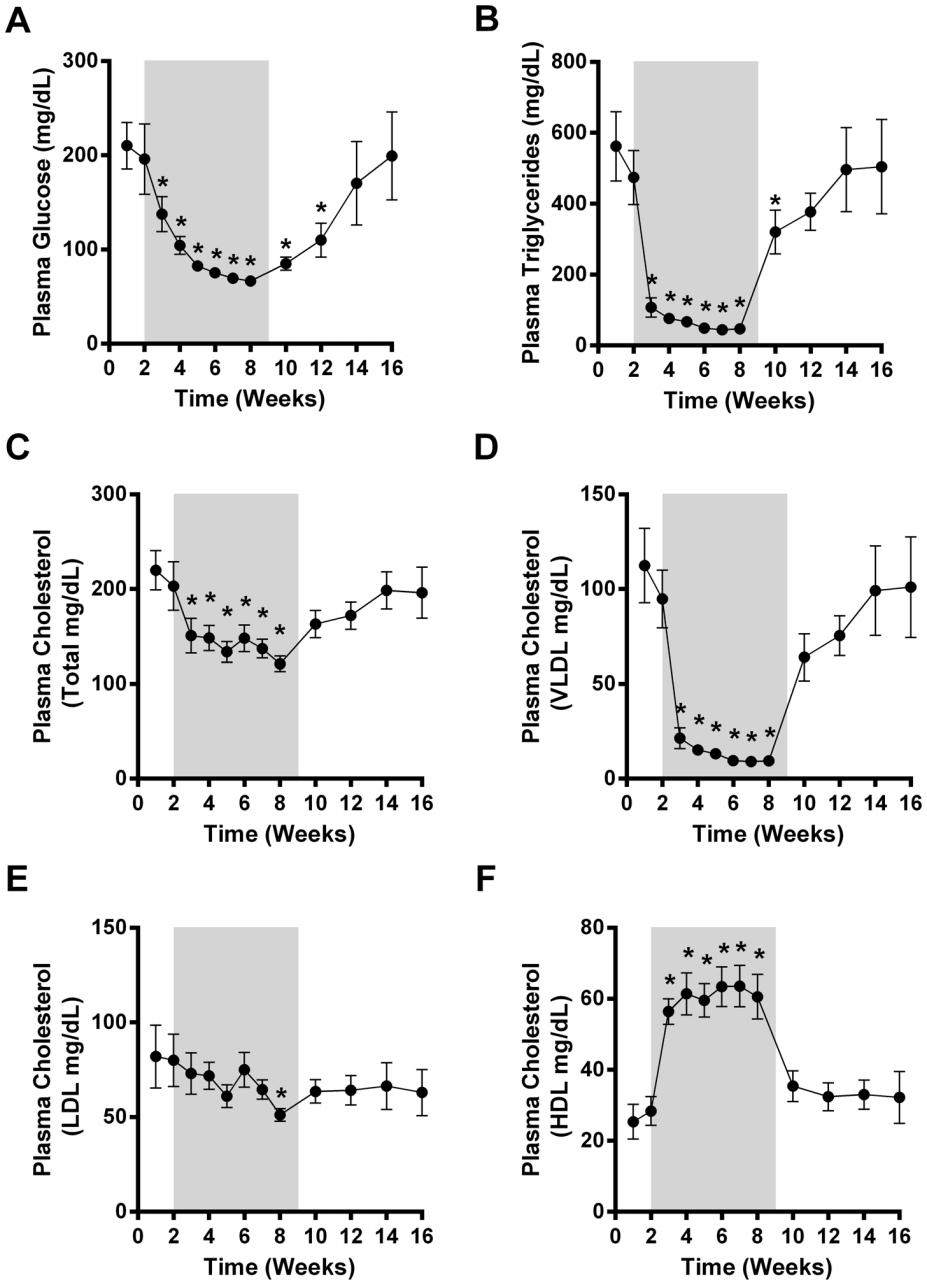
Weekly measurements of circulating glucose (A), triglycerides (B), total cholesterol (C), VLDL cholesterol (D), LDL cholesterol (E) and HDL cholesterol (F) throughout the duration of the study. Differences considered significant (p<0.05) are denoted by *.

**Figure 3:**
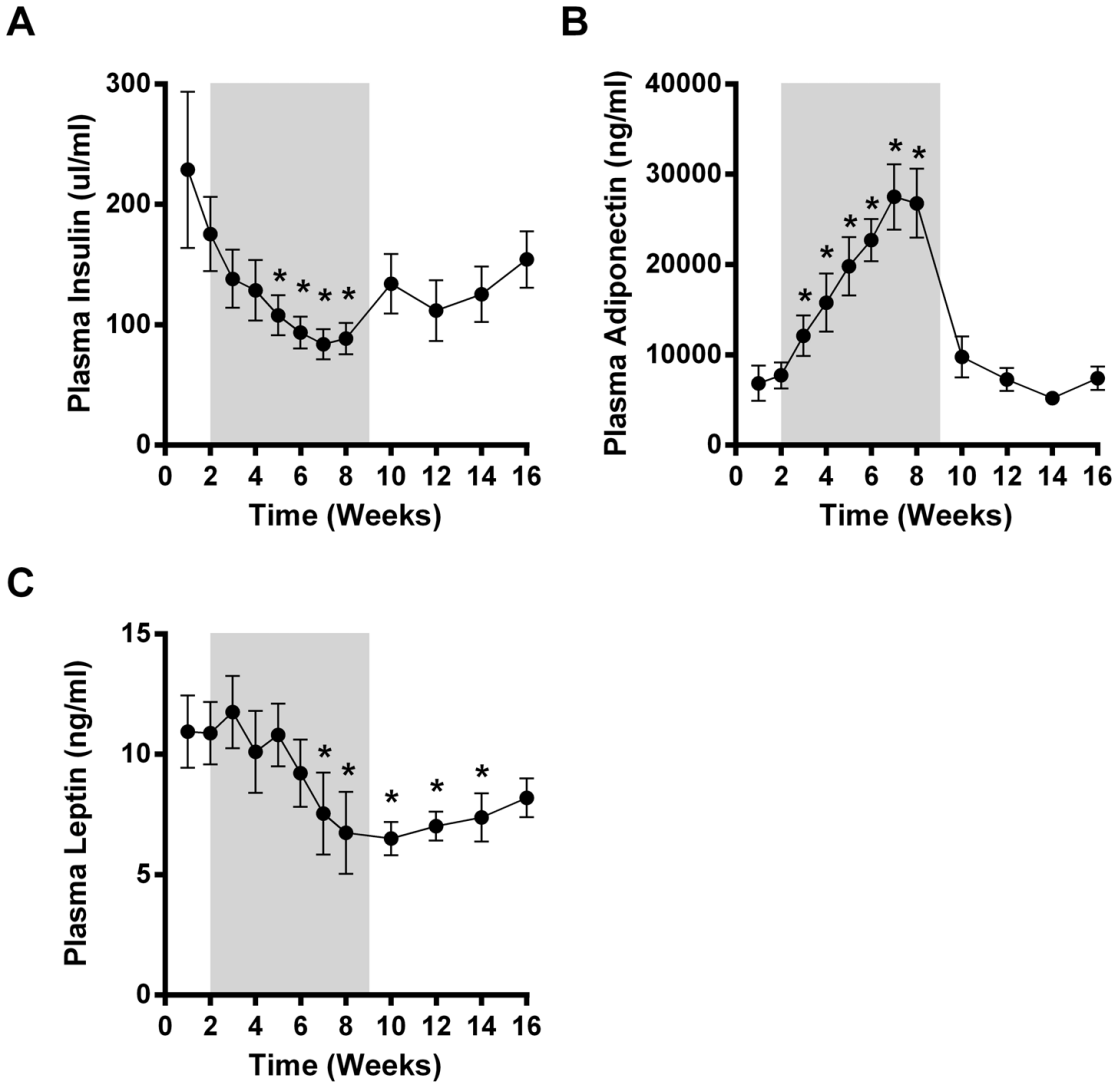
Prominent hormones such as insulin (A), leptin (B) and adiponectin (C) were found to be regulated by LY2405319. Differences considered significant (p<0.05) are denoted by *.

**Figure 4:**
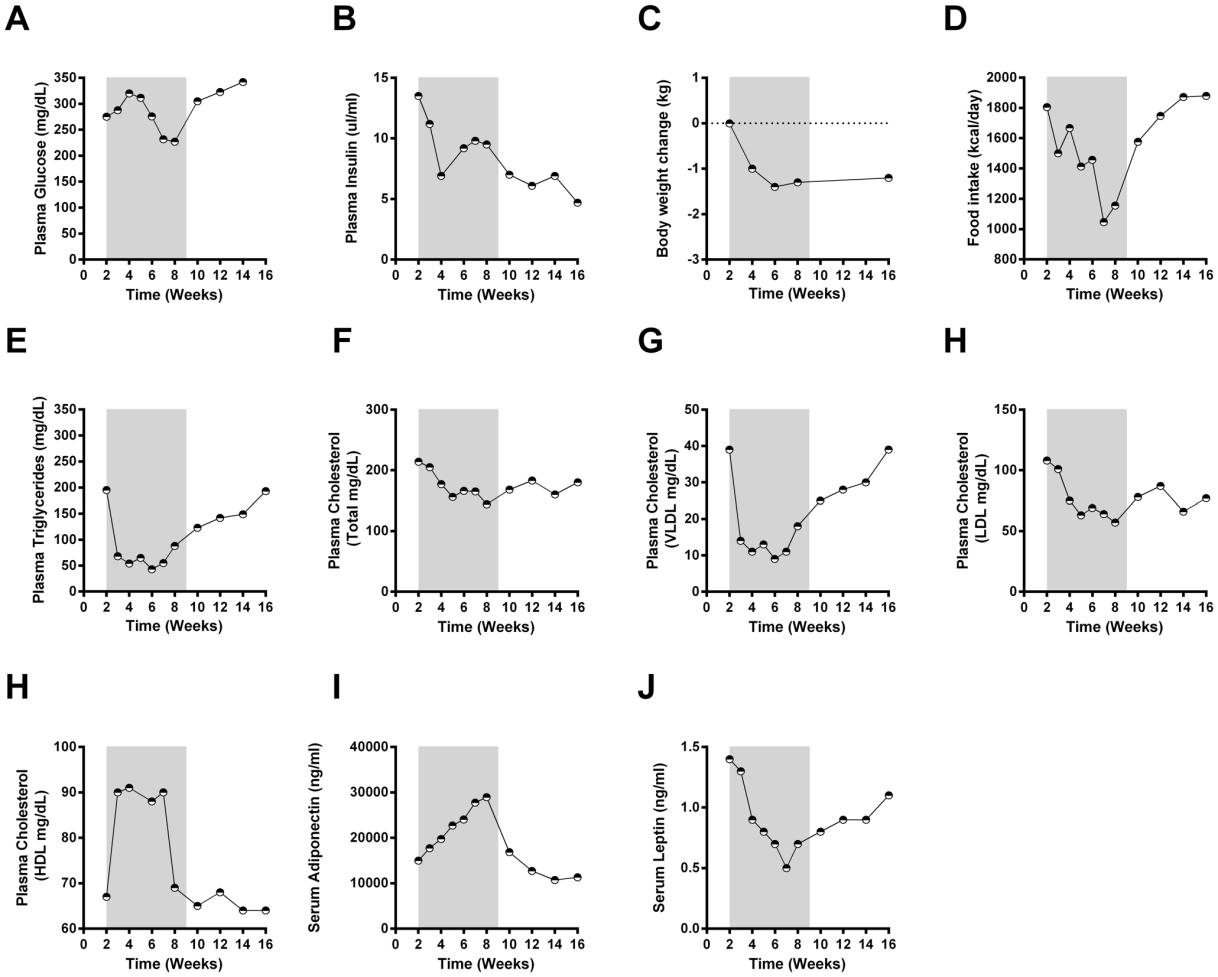
Data from J-15 are presented as a separate figure given more advanced disease state of this animal at study onset. As with the other animals we describe glucose (A) and insulin (B) in addition to body weight change (C), food intake (D) along with circulating levels of triglycerides (E), total cholesterol (F), VLDL cholesterol (G), LDL cholesterol (H) and HDL cholesterol (I). Furthermore, adiponectin (J) and leptin (K) were also determined. Differences considered significant (p<0.05) are denoted by *.

Prior to the lead in period we determined that one of the 6 animals selected for the study displayed significantly more advanced disease progression than the other members of the cohort, as evidenced by extremely low insulin levels in the context of overt hyperglycemia and low body weight as compared to the rest of the animals in the treatment cohort that were hyperinsulinemic and obese. However, to provide insight into FGF21 biology in the context of severe diabetic pathology we included this animal in the study and present some of the data obtained in this monkey separate to that of the remaining members of the cohort, both to highlight FGF21 effects in late stage disease and also to prevent distortion of the values reported for the other animals. All pharmacokinetic and immunogenicity data in Table 1 includes all 6 animals. Data in Figures 1, 2 and 3 represent the mean of 5 animals while the results in Figure 4 represent single case observations of the animal with advanced disease progression.

**Table 1:** Pharmacokinetics and Immunogenicity of LY2405319 in diabetic rhesus monkeys.

Treatment Period	Average AUC (0–24 h) ±SD (ng*h/mL)	LY-Reactive Antibody Range (Titer)
**Vehicle lead-in**	NC	<10 to 50
**3 mg/kg**	22080±3487	<10 to 250
**9 mg/kg**	68386±24064	50 to 1250
**50 mg/kg**	417156±127608	50 to >31250
**Follow-up (2 or 3** **wk)**	NC	250 to 6250
**Follow-up (4** **wk)**	NC	50 to 6250
**Follow-up (6** **wk)**	NC	50 to 2503
**Follow-up (8** **wk)**	NC	50 to 250

“ND” indicates values obtained were below the lower limit of detection of the assay (<200 pg/mL). “NC” indicates values were not calculated. “<10″ indicates that 1∶10 was the minimum required dilution of the antibody assay and antibody was not measured at this dilution. “>31250” indicates that the results were higher than the terminal titration of the sample.

### Blood Plasma Analyses

Blood glucose, fructosamine, and triglycerides (TGs) were determined by Antech Diagnostics (New York, NY). Fasting plasma insulin and glucagon were measured using enzyme-linked immunosorbent assay (ELISA) kits from Linco Diagnostic Services, Inc. (St. Charles, MO). Total cholesterol, low-density lipoprotein cholesterol (LDL-c), and high-density lipoprotein cholesterol (HDL-c) were measured using a Hitachi 912 clinical chemistry analyzer. Cytokine analysis was done by Rules-Based Medicine, Inc. (Austin, TX) using human antigen Multi-Analyte Profiles (MAP) kits. All clinical chemistry, lipid composition, cytokine, and other analyses presented in this report were performed on the blood samples collected 24 h after the last dose unless stated otherwise.

### LY Immunoreactivity

Serum samples were analyzed for concentrations of immunoreactive LY protein using anti-LY antibodies by the sandwich ELISA method. Samples were analyzed in duplicate and, when appropriate, at multiple dilutions.

### LY Immunogenicity

LY immunogenicity was assessed using a qualitative ELISA for the detection of LY-reactive antibodies in rhesus monkey serum samples with a detection limit of 1,150 relative ng/mL and LY tolerance of ≤1.0 µg/mL.

### Statistical Analysis

Data are presented as mean ±SEM. Data were analyzed using repeated-measures analysis applied for multi-dose designs utilizing post hoc analysis (Tukey). Differences were considered significant when p = <0.05.

## Results

### Pharmacokinetics of LY in Diabetic Rhesus Monkeys

Exposures to LY following its chronic subcutaneous administration to diabetic rhesus monkeys were evaluated by sandwich ELISA. LY levels were measured in fasting plasma samples collected weekly during the lead in and treatment phase. The mean plasma concentration of LY from each dosing phase increased in a dose dependent manner from 3 to 50 mg/kg (Table 1). There was no significant buildup of LY in plasma, as evidenced by the proportional increase in exposure at each dose escalation (Table 1).

### LY Immunogenicity

All 6 animals demonstrated positive anti-LY2405319 antibody titers during the study. LY-reactive antibodies were measured in serum samples collected during the vehicle lead-in and 1–2 days following the final dose for each treatment period. Additional samples were collected during week 2 and 3 of the follow-up period, and during weeks 4, 6, and 8 of the follow-up period. Treatment-emergent anti-LY immunoglobulins were detected in all 6 monkeys with titers ranging from 10 to >31,250 (Table 1). The reported titers may be underestimated due to the presence of residual serum concentrations of LY (>1.0 µg/mL).

### Body Weight Loss and Food Intake Reduction

Similar to the effects observed with administration of wild type FGF21 in rodents [7]–[9] and in primates [12], [15], all monkeys experienced progressive weight loss during each dosing phase amounting to 12.5%–23.6% reduction from baseline body weight over the 7 weeks of treatment (Figure 1A). During the washout phase, all animals began to gain weight, although in spite of rebound weight gain animals still weighed less at the end of the washout period than prior to LY dosing (Figure 1A).

Food consumption was assessed daily and converted to weekly caloric intake. Following two weeks of LY treatment food intake was dramatically suppressed when compared to basal values similar to the results of the previous studies with FGF21 in this species [12], [15]. This reduction in caloric intake persisted until the final week of compound administration and was dose dependent in nature (Figure 1B). During the washout phase, the food intake initially returned to the baseline level then transiently increased over pretreatment intake suggesting a compensatory/rebound response following the cessation of the LY treatment (Figure 1B). The reduction in calorie consumption during the LY administration phase likely contributed to the observed body weight lowering effect.

### Glycemic Effects of Chronic LY Administration

Prior to LY administration, mean baseline plasma glucose was above 200 mg/dL (Figure 2A), approximately three fold higher than normal and significantly elevated relative to the diagnostic criteria established for type 2 diabetes in this species [16]. LY treatment decreased mean non-fasted blood glucose concentrations in a rapid manner, from an overtly diabetic level to a complete normalization that was highly sustainable throughout the entire dosing phase (Figure 2A). Importantly, no incidents of hypoglycemia were noted in any animal at any dose of LY even though large reductions in circulating glucose concentrations were observed during the treatment period. Upon cessation of compound administration, blood glucose increased over time, reaching at the end of the washout period concentrations similar to those in the lead-in phase (Figure 2A).

### LY Lowers Systemic Lipids in an FGF21 Like Manner

Mean fasting plasma triglyceride concentrations were significantly reduced during LY administration, with near-maximal efficacy already observed after only 1 week of treatment at the lowest dose, and only slight additional reductions with higher doses during the remainder of the treatment period (Figure 2B). Thus, after 7 weeks of LY therapy, circulating triglycerides were lowered by 87% relative to baseline values. LY also had a significant and positive impact on the circulating cholesterol profile in the monkeys with its total content decreased by 39% at the end of dosing (Figure 2C). When broken down by fraction there was a significant reduction in VLDL levels which were decreased by approximately 87% at the final dosing time point (Figure 2D). LDL levels were also lower at the final time point of the treatment period (Figure 2E). Importantly, the HDL fraction was significantly elevated throughout by all doses of LY with a 77% increase at the end of the LY administration (Figure 2F).

### LY Regulates Circulating Hormones and Adipokines

Recently our group and others have reported FGF21-induced regulation of the circulating levels and improved total body sensitivity to a multitude of other metabolic factors such as insulin, leptin and adiponectin highlighting one of the major mechanisms of FGF21 *in vivo* action [7], [9], [15], [17]. In this study with the use of LY in diabetic monkeys we are able to replicate these earlier findings. Concomitant with the reductions in plasma glucose, circulating insulin levels were robustly reduced, with complete normalization observed in the final week of treatment (Figure 3A). Adiponectin was significantly elevated already after 1 week of LY therapy and continued to increase throughout the treatment period. Following cessation of dosing, adiponectin levels dropped rapidly returning to basal values after only 2 weeks of a follow-up phase. Plasma leptin levels trended to be reduced during both the 3 mg/kg and 9 mg/kg dosing periods and were significantly lower for the final 3 weeks of LY administration at the 50 mg/kg dose, then trended up but still remained lower for the duration of the follow-up period.

### LY is Partially Efficacious in An Animal with Advanced Diabetes Disease Progression

To date, it has not been determined whether FGF21 based therapy, often deemed to correct hyperglycemia via an insulin sensitization mechanism [6], remains efficacious with regard to glucose endpoints in the context of low insulin levels. Therefore, in addition to the overtly hyperglycemic and hyperinsulinemic animals in the primary study group we also evaluated the pharmacologic response to LY in one other animal (hereby referred to as J-15). J-15 presented with extremely low pre-treatment insulin levels (14 µU/mL), significant hyperglycemia (275 mg/dL, fed glucose) and low body weight (<10 kg at study intake) representative of a more advanced stage of diabetes disease as compared to 5 other monkeys in the main cohort. Treatment of J-15 with low and intermediate doses of LY did not lead to serum glucose reduction, and only at the highest 50 mg/kg dose glucose lowering was observed. This effect was however fairly attenuated and did not reach normalization or glycaemia (Figure 4A) as compared to the LY efficacy toward glucose lowering in the primary study group (Figure 2A). Importantly, there was also only a slight modulation of insulin levels in J-15 throughout the entire dosing period (Figure 4B).

Interestingly, and in spite of the fact that J-15 had lower body weight at the beginning of the study, LY still remained potently efficacious to induce body weight loss (Figure 4C). This body weight lowering is thought to be in part due to a dramatic reduction in food intake during the entirety of the drug administration period, similar to that seen in the cohort as a whole (Figure 4D). In contrast to the attenuated ability to impact glycaemia and insulin, there was no loss of LY efficacy in J-15 toward lipid endpoints; the effects were observed with triglycerides (Figure 4E), total cholesterol (Figure 4F), VLDL (Figure 4G), LDL (Figure 4H) and HDL (Figure 4I). With regard to their magnitude, the changes in lipid profiles in J-15 resembled those observed in the other monkeys. Furthermore, plasma adiponectin levels in J-15 were elevated by all LY doses and returned to baseline rapidly at the cessation of treatment (Figure 4J). Finally, circulating leptin during the lead in phase was low in this animal likely due to J-15′s low adiposity; however, in line with the other animals, it was further reduced by LY treatment (Figure 4K).

## Discussion

Non-human primates are thought to develop type 2 diabetes in a very similar manner to that observed in humans and thus offer an ideal test system for testing of anti-diabetic therapeutics [16]. Indeed, our group and others have reported significant potency of both native FGF21 [12], [15] and FGF21 variants [12] in this model. Previously, we have demonstrated that our LY retains equivalent *in vivo* efficacy in rodent models of obesity and diabetes with no significant differences in the mechanism of action when compared to wild type FGF21 [14]. Thus, given the generally exceptional translational quality of the experimental data obtained in rhesus monkeys that developed diabetes in a natural way [16], we chose to evaluate our FGF21-based candidate, LY, in this model.

Importantly, we show here that LY displays therapeutic characteristics similar to wild type FGF21 in the rhesus monkey. As with FGF21 administration, LY was able to rapidly and robustly improve and maintain tight glycemic control within the normal range over the course of the treatment while also delivering dramatic reductions in both circulating lipids and body weight. Together, these findings confirm that our engineered variant of the native FGF21 protein, LY, demonstrates therapeutic characteristics similar to FGF21 in non-human primates. The maximal efficacy of LY and wild type FGF21 are indeed similar which is likely due to the fact that neither FGF21 nor LY are able to lower glucose beyond the normal range in these species (Figure 2A, [15]). Nevertheless, if one compares the time taken to reach significantly meaningful glucose lowering, LY achieves glucose normalization in approximately 1 week of treatment (Figure 2) while in our previous study with the native FGF21 protein this effect took approximately 4 weeks to attain significance [15]. Given that higher LY doses were utilized in this study compared to our previous experiment [14], this differential in the onset of correction of hyperglycemia raises the possibility that greater doses of FGF21 and/or use of FGF21 variants with improved potency may offer a more rapid and sustainable therapeutic response. It is noteworthy that despite the development of antibodies to LY, the pharmacologic effects were sustained through the end of the 7-week treatment period suggesting that LY antibodies were not neutralizing in nature.

Recently we and others have reported that the metabolic effect of FGF21 is heavily reliant on engagement of the FGFR1/KLB complex in white adipose tissue [10], [18]. Activation of this receptor complex in fat leads to profound changes in circulating adipokines indicative of increased sensitivity to these factors in their target tissues [10], [19], [20]. Here we demonstrate that treatment with LY also leads to a rapid and robust induction of adiponectin, a known insulin sensitizer [21]. Importantly, we show in the present study that this increase in plasma adiponectin is not driven by modulation of either adiposity or food intake as the massive induction of adiponectin returns to normal prior to significant changes in weight or caloric intake. Thus, LY, as well as native FGF21, is a potent *in vivo* adiponectin secretagogue. Given that FGF21 is unable to lower glucose in adiponectin null mice [22], [23] one can surmise that the increase in adiponectin is explicitly required for FGF21s glycemic effect. When coupled with the insulin lowering phenotype observed in the monkeys and the fact that the induction of adiponectin is also present in higher mammals these data serve as an additional indicator of potential primate-to-human translatability of the glucose lowering effect observed with LY.

While caution must be exercised due to the single case nature of the data, the fact that LY remained partially efficacious in an animal at the late stage of disease development opens the possibility that FGF21 based therapy may have therapeutic utility in patients with well advanced type 2 diabetes. Intriguingly, and even though the glycemic efficacy of LY did appear to be partially blunted, adiponectin levels in J-15 were increased robustly and to a similar degree as in the other 5 obese monkeys (Figure 3B vs. Figure 4I]. Thus, adiponectin appears to be necessary but not entirely sufficient to trigger robust FGF21-driven glucose lowering in an animal at an advanced stage of disease progression. Further mechanistic insight into this dichotomy likely centers around the lack of meaningful insulin production in J-15 (Figure 4B), as without appropriate plasma levels of insulin the glycemic effect of adiponectin, a sensitizer to insulin action, is unlikely to occur. Nevertheless, beyond impaired glucose lowering, the ability of LY to lower plasma lipids and body weight was fully retained in J-15 as evidenced by similar changes in these parameters when compared to those observed in the larger cohort (Figure 1 & 2 vs. Figure 4). This further supports the idea of FGF21 being a truly pleiotropic factor *in vivo,* able to induce a multitude of the metabolic effects in a parallel manner via an array of diverse adipose centric mechanisms [17].

### Conclusions

Our data show that the FGF21 based clinical candidate, LY, is robustly efficacious in diabetic rhesus monkeys where it is able to induce the full gamut of FGF21 biology. Chronic administration of the compound led to dramatic improvements in both serum glucose and lipids in addition to robust effects on food intake and body weight. Furthermore, in an animal with late stage diabetes, FGF21 therapy retained the majority of its efficacy with attenuation of only glycemic aspects of FGF21-mediated pharmacology. In conclusion, this work provides further evidence to support the investigation of FGF21 as a potential multifaceted “glucose plus” agent and supports further discovery and development of FGF21 based therapies.
